# Sex-specific responses to winter flooding, spring waterlogging and post-flooding recovery in *Populus deltoides*

**DOI:** 10.1038/s41598-017-02765-2

**Published:** 2017-05-31

**Authors:** Ling-Feng Miao, Fan Yang, Chun-Yu Han, Yu-Jin Pu, Yang Ding, Li-Jia Zhang

**Affiliations:** Institute of Tropical Agriculture and Forestry, Hainan University, Haikou, Hainan 570228 P. R. China

## Abstract

Winter flooding events are common in some rivers and streams due to dam constructions, and flooding and waterlogging inhibit the growth of trees in riparian zones. This study investigated sex-specific morphological, physiological and ultrastructural responses to various durations of winter flooding and spring waterlogging stresses, and post-flooding recovery characteristics in *Populus deltoides*. There were no significant differences in the morphological, ultrastructural and the majority of physiological traits in trees subjected to medium and severe winter flooding stresses, suggesting that males and females of *P*. *deltoides* were winter flooding tolerant, and insensitive to winter flooding duration. Males were more tolerant to winter flooding stress in terms of photosynthesis and chlorophyll fluorescence than females. Females displayed greater oxidative damage due to flooding stress than males. Males developed more efficient antioxidant enzymatic systems to control reactive oxygen species. Both sexes had similarly strong post-flooding recovery capabilities in terms of plant growth, and physiological and ultrastructural parameters. However, Males had better recovery capabilities in terms of pigment content. These results increase the understanding of poplars’s adaptation to winter flooding stress. They also elucidate sex-specific differences in response to flooding stress during the dormant season, and during post-flooding recovery periods.

## Introduction

Riparian forests often experience a wide range of flooding or waterlogging conditions, which can cause declines in growth and even the death of certain plant species. Furthermore, winter flooding events are common in some rivers and streams due to the artificial water level regulation associated with dams^1, 2^. For example, to operate China’s Three Gorges Dam at full capacity, the water level of the Three Gorges Reservoir was artificially regulated at a winter maximum of 175 m for energy generation, and a summer minimum of 145 m for flood control^1, 2^. Thus, the hydrological regimes brought about by dams can be the opposite of the river’s natural flood rhythms. This can cause some riparian forests to suffer winter flooding stress while enduring floods of various durations and depths. Flood tolerance varies greatly with plant species and genotype, the sex of dioecious plants, plant age, flooding duration and depth, and flooding season^3–9^. Previous studies have confirmed that *Populus deltoides* is flood-tolerant, and can spread widely in European and North American riparian and floodplain zones^5, 6, 10–12^. *P. deltoides* is not naturally occurring in China, and must be imported from North America. It is recognized as a desirable tree species for the construction of riparian-protective forests in China because of its fast growth and strong tolerance to waterlogging stress^5, 6^. *P. deltoides* belongs to Sect. *Aigeiros*. Revegetation activities in the water level fluctuation zone of the Three Gorges Reservoir have demonstrated that Sect. *Aigeiros* poplars, including *P. nigra* and *P*. × *canadensis*, could be suitable for the construction of riparian-protective forests^1, 2^. However, the mechanisms by which *P. deltoides* adapts to winter flooding and recovers afterward are still unknown.

Previous studies have focused on the phenotypic plasticity and adaptive plasticity of poplars in response to summer flooding or waterlogging stresses during the plant growth stage of development^4–8^. Poplars are deciduous species. Deciduous plants’ physiological and molecular properties are markedly different during the dormant and growing seasons^13–15^. However, few studies have investigated the responses of poplars to winter flooding stress during the dormant season. The morphological, physiological and ultrastructural responses of poplars to winter flooding stress during dormancy, and during post-growth recovery, remain poorly understood.

*Populus* spp. is a dioecious species. Previous studies have established the sex-specific morphological, physiological, and biochemical characteristics of poplars in response to environmental stress^16–21^. Different sexes of poplars might employ different strategies to cope with abiotic stress, and that males possess a better self-protection mechanism than females. Our previous studies have also demonstrated that *P. deltoides* males develop better cellular defense mechanisms against waterlogging stress than females, making males less susceptible^6^. However, Juvany and Munné-Bosch^22^ reviewed responses to abiotic and biotic stresses and suggested that general conclusions about sex-related stress tolerance in plants were not possible. For example, Nielsen *et al*.^4^ and Rood *et al*.^8^ suggested that female *P. angustifolia* were more flood-tolerant than males, and that females could be more successful in lower, more flood-prone sites. However, Letts *et al*.^7^ reported that there were no significant differences in the photosynthetic gas exchange, leaf reflectance, chlorophyll fluorescence or photosynthetic water-use efficiency of female and male *P. angustifolia*. In addition, comparisons of the sex-dependent responses of poplars to abiotic stress have largely been conducted in summer during the fast growth stage. Little study has occurred regarding responses to winter flooding during dormancy and post-growth recovery. Consequently, the physiological mechanisms underlying sex-related differences in winter flooding stress responses remain poorly understood.

The present study investigated sexual dimorphism in *P. deltoides* during winter flooding, waterlogging, and post-flooding recovery. The main research questions were as follows. (1) How does winter flooding stress affect *P. deltoides*? (2) What are the responses to different durations of winter flooding? (3) Do these responses differ between the sexes? (4) Do winter flooding-stressed trees recover to normal levels in terms of morphological, physiological and ultrastructural traits? (5) Are there sexual differences in recovery characteristics? To answer these questions, we measured morphological, physiological and ultrastructural variations to reveal gender-related responses to winter flooding stress and post-flooding recovery.

## Results

### Comparative analysis of survival rates and morphological traits

Generally, all flooding-stressed *P. deltoides* seedlings survived, and epicormic shoot germination from nodes and new leaf emergence occurred almost simultaneously with controls. With increasing flooding duration, significant visible damages, such as mortality, leaf chlorosis, leaf necrosis, or leaf abscission were not observed during the plant growth stage of development. Morphological adaptations such as hypertrophied lenticels, aerenchyma tissues and adventitious roots, which often occur when plants are exposed to summer waterlogging stress, did not appear in the present study.

**Figure 1:**
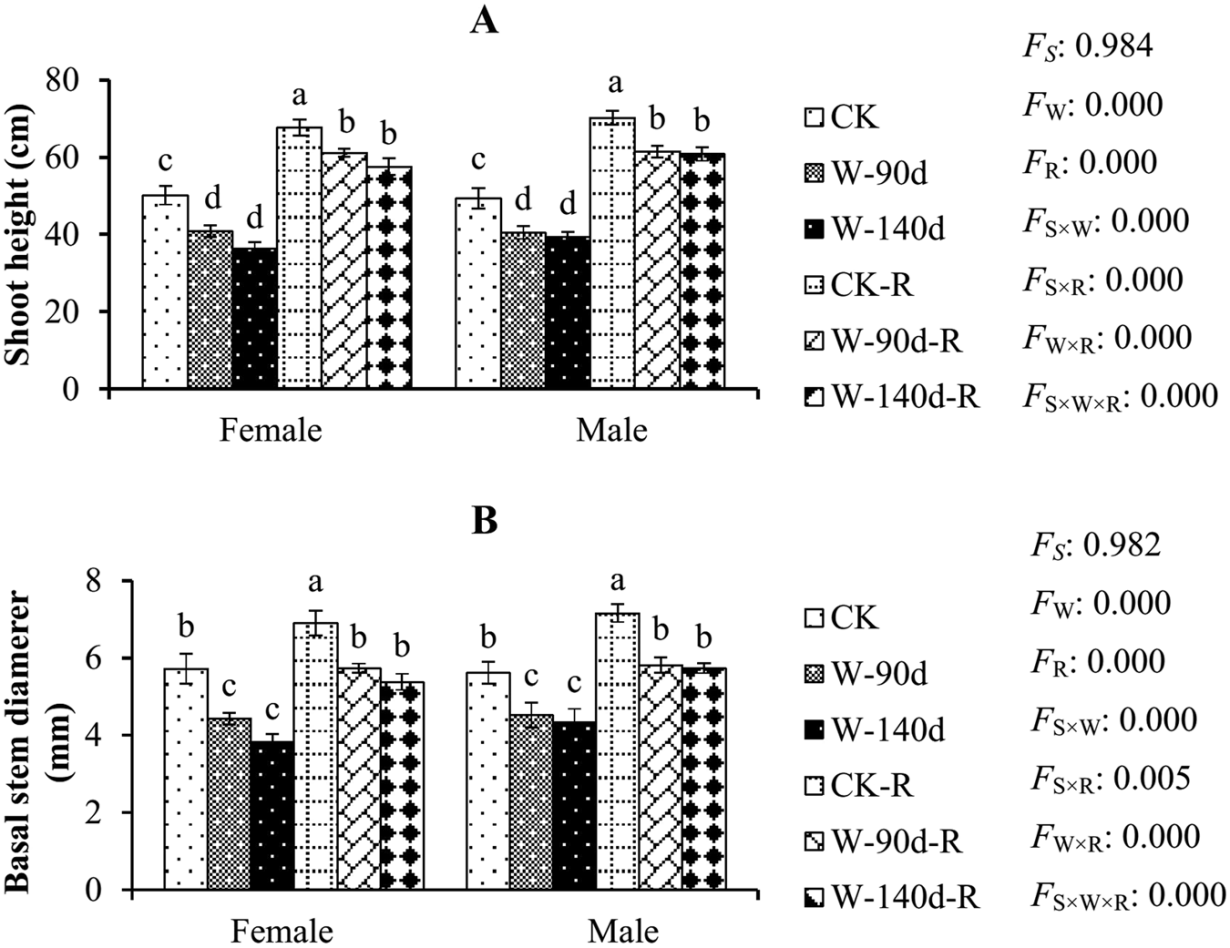
Morphological variations in male and female *P. deltoides* under winter flooding stress and post-flooding recovery. CK, control treatment; W, flooding and waterlogging stress treatment; R, recovery after waterlogging stress. Values are means ± SE (*n* = 5). Letters above the columns indicate significantly differences at *P* < 0.05 according to Duncan’s test. The significance values of the factorial analysis (ANOVA) for: *F*_*S*_, *F*_W_, and *F*_R_ refer different effects of species, watering, and recovery growth, respectively; *F*_S×W_, *F*_S×R_, *F*_W×R_, and *F*_S×W×R_ refer species × watering effects, species × recovery growth effects, watering × recovery growth effects, and species × watering × recovery growth effects, respectively.

When flooding was combined with waterlogging, shoot height (Fig. 1A) and basal stem diameter (Fig. 1B) were significantly inhibited in comparison to controls. Greater inhibition was observed with increasing flooding duration. Significant differences were not found between the two flooding treatments (W-90d and W-140d). In addition, significant differences in the shoot height and basal stem diameter of females and males were not found within each flooding treatment.

During the recovery stage, the shoot heights and basal stems of flooding stressed females and males both recovered well. For example, shoot height growth rates in females and males were 38.8% and 42.4% in CK, respectively, whereas they were 49.8% and 51.8% in W-90d, and 57.9% and 54.4% in W-140d during the recovery stage. The basal stem growth rates in females and males were 21.0% and 27.6% in CK, respectively, whereas they were 29.3% and 28.8% in W-90d, and 40.8% and 32.0% in W-140d during the recovery stage. In addition, significant differences were not found between the two post-flooding recovery treatments (W-90d-R and W-140d-R). In addition, there were no significant differences in shoot height and basal stem during the recovery stage between females and males within each post-flooding recovery treatment.

### Winter flooding stress affected the physiological traits of two-year-old *P. deltoides* trees at an early stage of development

**Figure 2:**
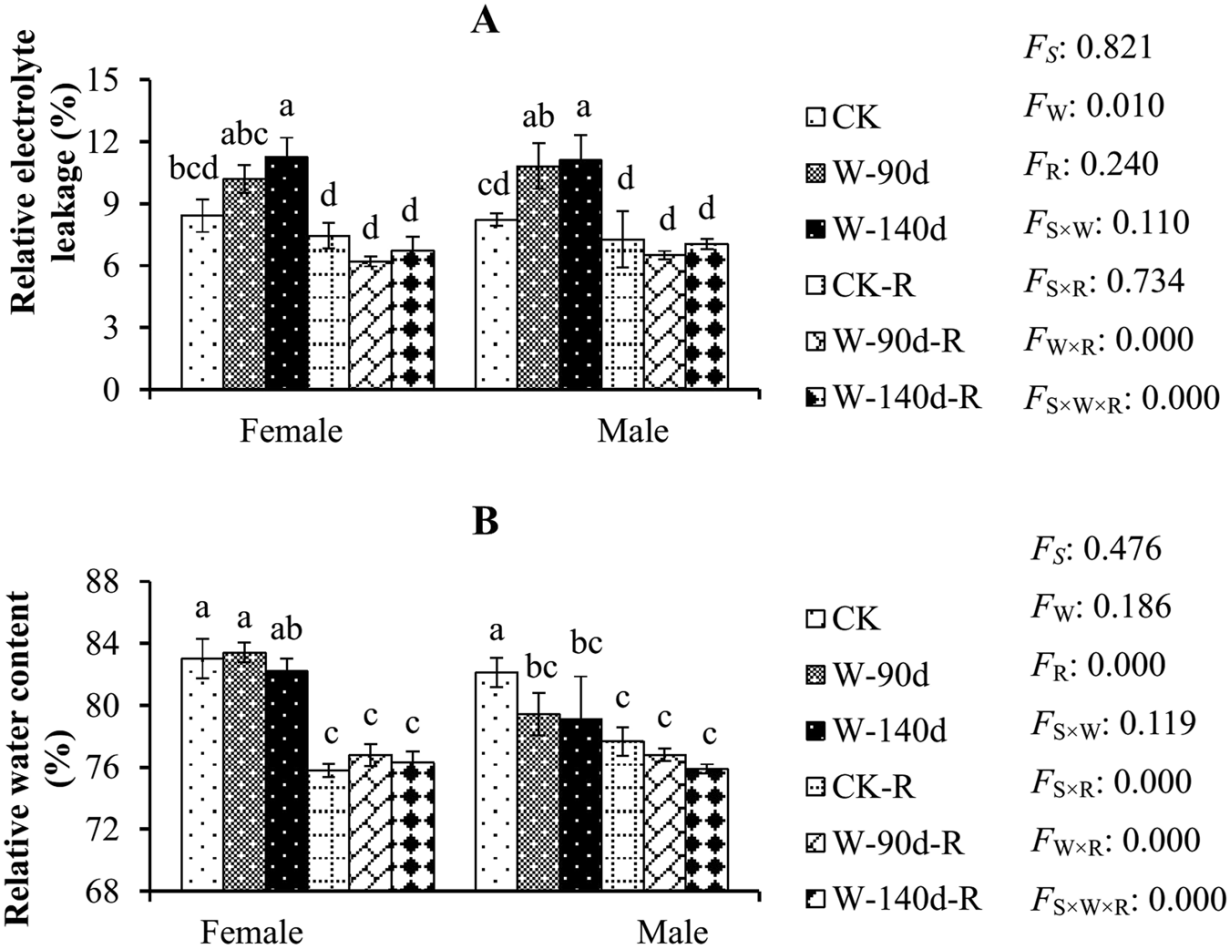
Maximum efficiency of PSII (*Fv/Fm*), effective quantum yield of PSII (*Yield*), photosynthetic electron transportation rate *(ETR*), photochemical quenching coefficient (*qP*), and non-photochemical quenching coefficient (*qN*) in male and female *P. deltoides* under winter flooding stress and post-flooding recovery. CK, control treatment; W, flooding and waterlogging stress treatment; R, recovery after waterlogging stress. Values are means ± SE (*n* = 5). Letters above the columns indicate significantly differences at *P* < 0.05 according to Duncan’s test. The significance values of the factorial analysis (ANOVA) for: *F*_*S*_, *F*_W_, and *F*_R_ refer different effects of species, watering, and recovery growth, respectively; *F*_S×W_, *F*_S×R_, *F*_W×R_, and *F*_S×W×R_ refer species × watering effects, species × recovery growth effects, watering × recovery growth effects, and species × watering × recovery growth effects, respectively.

**Figure 3:**
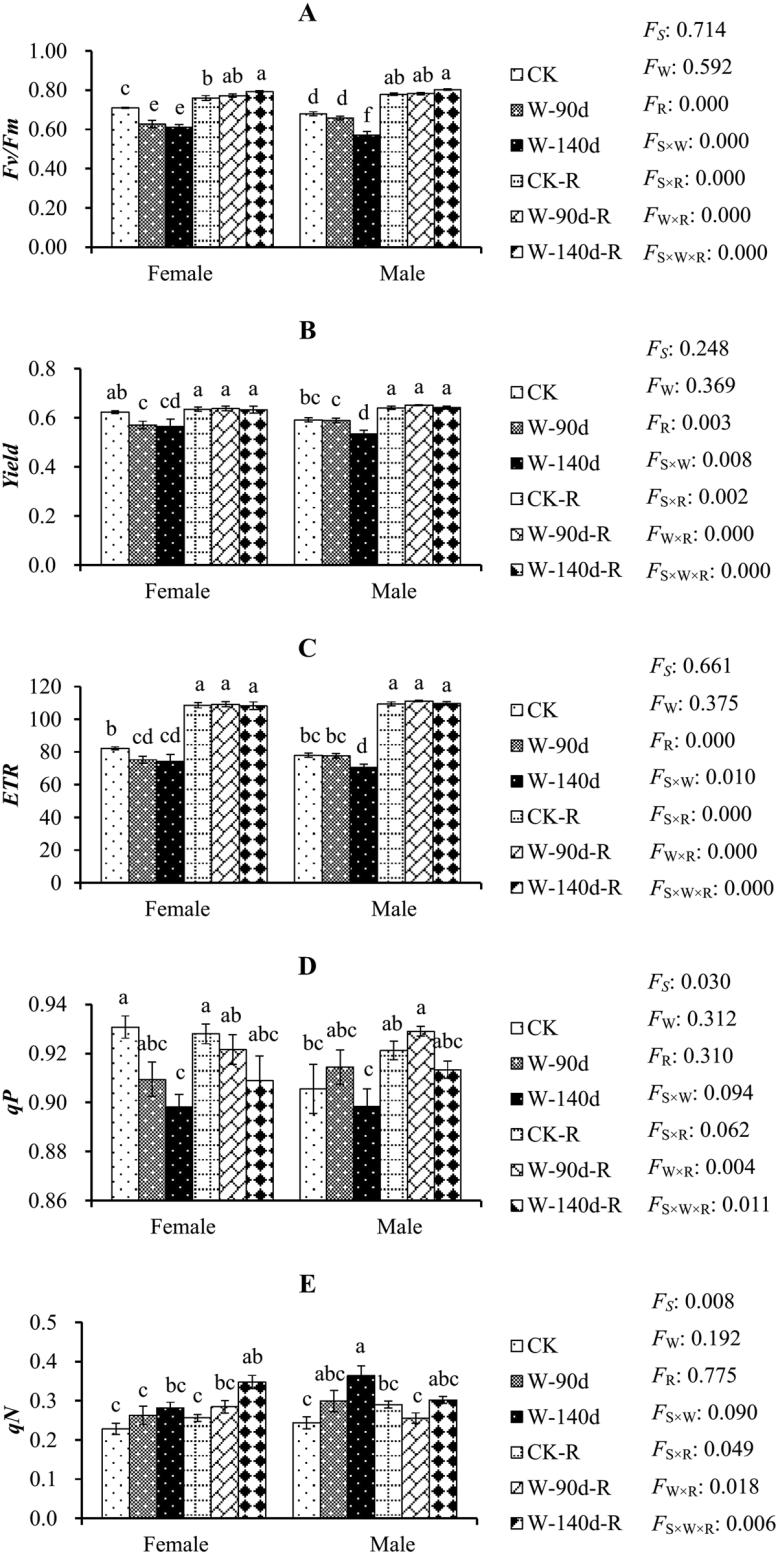
The relative electrolyte leakage (REL) and relative water content (RWC) variations in male and female *P. deltoides* under winter flooding stress and post-flooding recovery. CK, control treatment; W, flooding and waterlogging stress treatment; R, recovery after waterlogging stress. Values are means ± SE (*n* = 5). Letters above the columns indicate significantly differences at *P* < 0.05 according to Duncan’s test. The significance values of the factorial analysis (ANOVA) for: *F*_*S*_, *F*_W_, and *F*_R_ refer different effects of species, watering, and recovery growth, respectively; *F*_S×W_, *F*_S×R_, *F*_W×R_, and *F*_S×W×R_ refer species × watering effects, species × recovery growth effects, watering × recovery growth effects, and species × watering × recovery growth effects, respectively.

**Figure 4:**
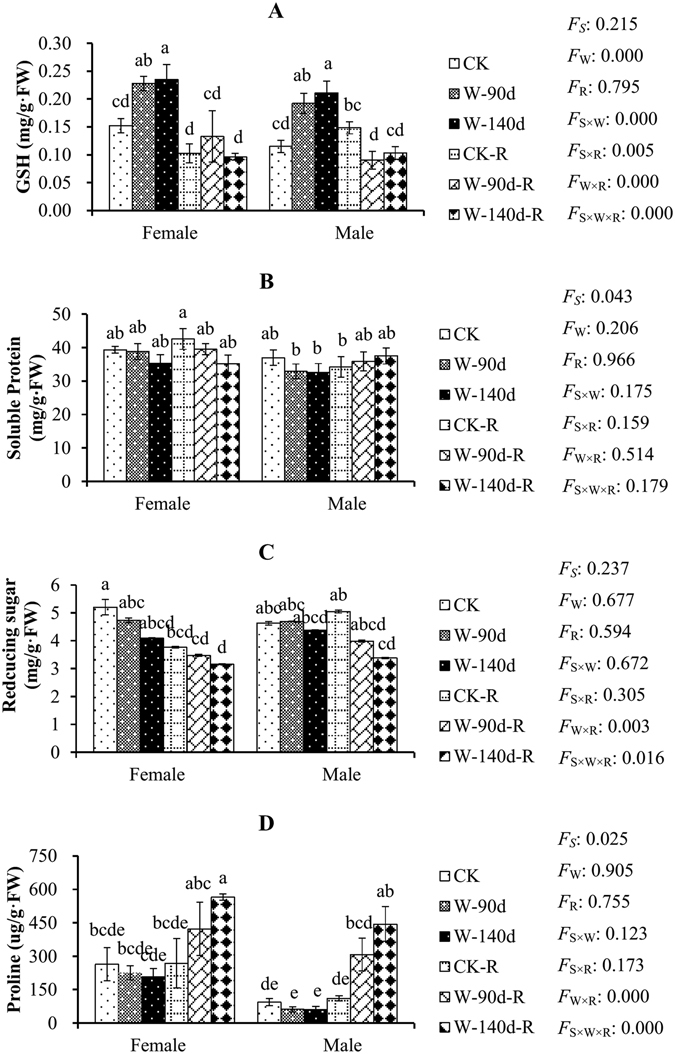
Glutathione (GSH), soluble protein, reducing sugar and free proline content variations in male and female *P. deltoides* under winter flooding stress and post-flooding recovery. CK, control treatment; W, flooding and waterlogging stress treatment; R, recovery after waterlogging stress. Values are means ± SE (*n* = 5). Letters above the columns indicate significantly differences at *P* < 0.05 according to Duncan’s test. The significance values of the factorial analysis (ANOVA) for: *F*_*S*_, *F*_W_, and *F*_R_ refer different effects of species, watering, and recovery growth, respectively; *F*_S×W_, *F*_S×R_, *F*_W×R_, and *F*_S×W×R_ refer species × watering effects, species × recovery growth effects, watering × recovery growth effects, and species × watering × recovery growth effects, respectively.

**Figure 5:**
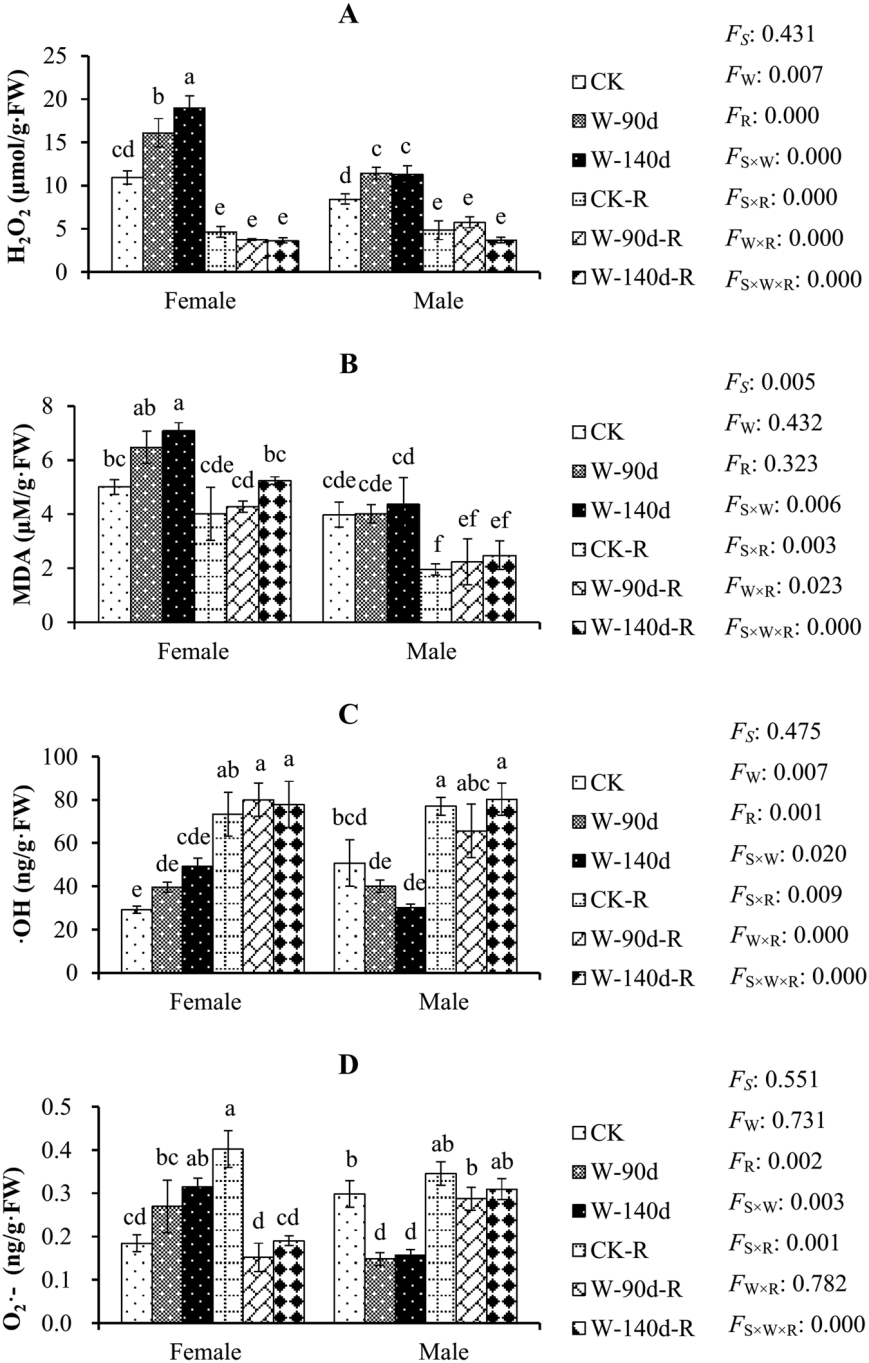
Hydrogen peroxide (H_2_O_2_), malondialdehyde (MDA), hydroxyl radical (·OH), and superoxide radical (O_2_^.−^), content variations in male and female *P. deltoides* under winter flooding stress and post-flooding recovery. CK, control treatment; W, flooding and waterlogging stress treatment; R, recovery after waterlogging stress. Values are means ± SE (*n* = 5). Letters above the columns indicate significantly differences at *P* < 0.05 according to Duncan’s test. The significance values of the factorial analysis (ANOVA) for: *F*_*S*_, *F*_W_, and *F*_R_ refer different effects of species, watering, and recovery growth, respectively; *F*_S×W_, *F*_S×R_, *F*_W×R_, and *F*_S×W×R_ refer species × watering effects, species × recovery growth effects, watering × recovery growth effects, and species × watering × recovery growth effects, respectively.

**Figure 6:**
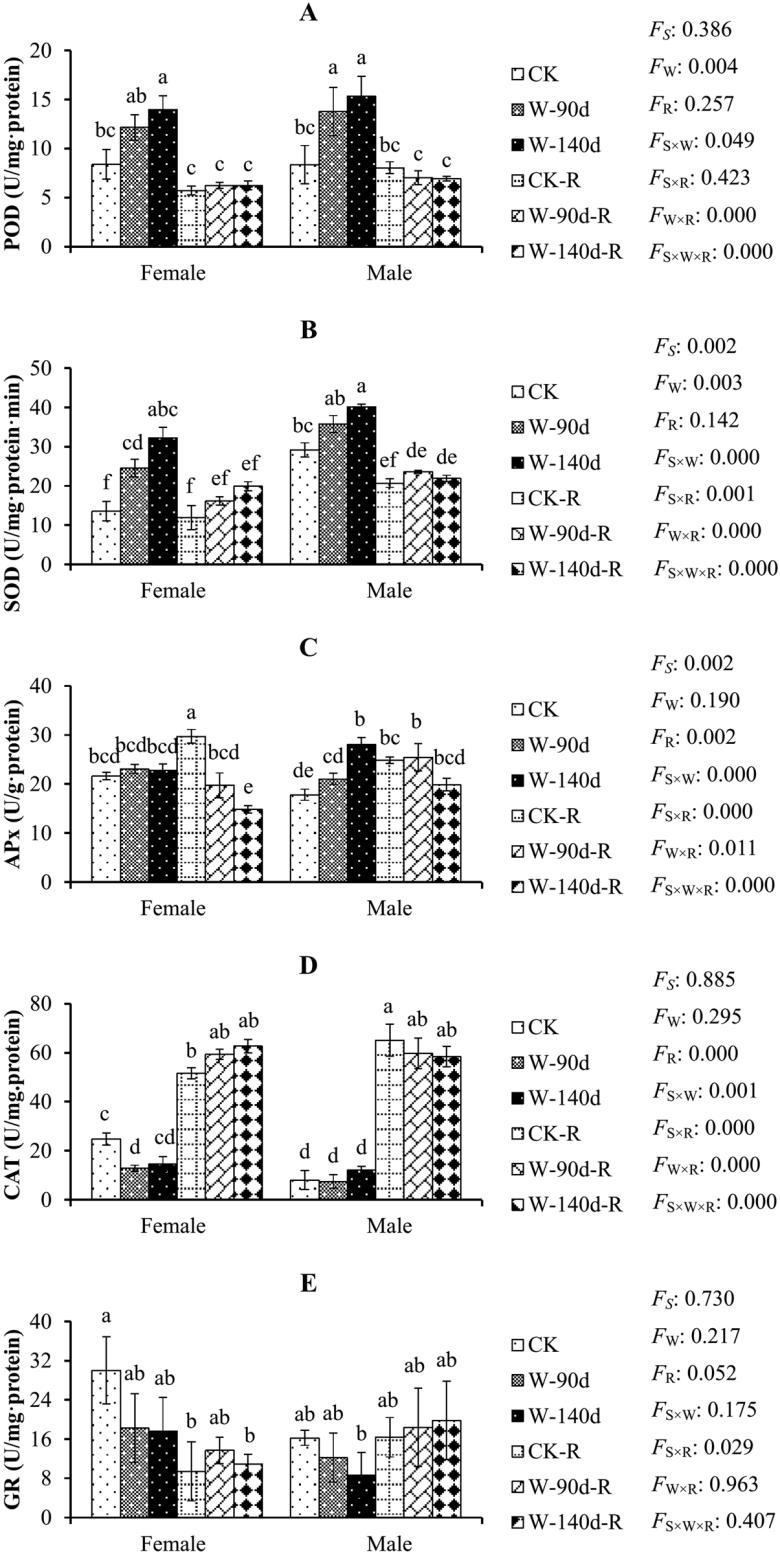
Peroxidase (POD), superoxide dismutase (SOD), ascorbate peroxidase (APx), catalase (CAT), and glutathione reductase (GR) activity variations in male and female *P. deltoides* under winter flooding stress and post-flooding recovery. CK, control treatment; W, flooding and waterlogging stress treatment; R, recovery after waterlogging stress. Values are means ± SE (*n* = 5). Values followed by different little letters are significantly different at *P < *0.05 according to Duncan’s test. The significance values of the factorial analysis (ANOVA) for: *F*_*S*_, *F*_W_, and *F*_R_ refer different effects of species, watering, and recovery growth, respectively; *F*_S×W_, *F*_S×R_, *F*_W×R_, and *F*_S×W×R_ refer species × watering effects, species × recovery growth effects, watering × recovery growth effects, and species × watering × recovery growth effects, respectively.

Severe flooding stress (W-140d) significantly decreased *A* (Table 1), *Fv/Fm* (Fig. 2A), *Yield* (Fig. 2B), and *ETR* (Fig. 2C), whereas it significantly increased REL (Fig. 3A), GSH content (Fig. 4A), H_2_O_2_ levels (Fig. 5A), and activities of POD (Fig. 6A) and SOD (Fig. 6B) in both sexes of *P. deltoides*. However, significant variations in RWC *E* (Table 1), *WUEi* (Table 1), *qP* (Fig. 2D), *qN* (Fig. 2E), (Fig. 3B), MDA (Fig. 5B), APx (Fig. 6C) were found only in either females or males, and significant variations in *Chl a* (Table 2), *Chl b* (Table 2), *Caro* (Table 2), *Total Chl* (Table 2), *Chl a*/*Chl b* (Table 2), *gs* (Table 1), soluble protein (Fig. 4B), reducing sugar (Fig. 4C), proline (Fig. 4D), ·OH (Fig. 5C), CAT (Fig. 6D), and GR (Fig. 6E) were absent in both sexes of *P. deltoides* exposed to W-140d conditions (Supplementary Table 1).

**Table 1:** Net photosynthetic rate (*A*), transpiration (*E*), stomatal conductance (*gs*), intercellular CO_2_ concentration (*Ci*) and intrinsic water use efficiency (*WUEi*) variations in male and female *P. deltoids* under winter flooding stress and post-flooding recovery.

Species	Treatment	*A* (μmol · m^−2^s^−1^)	*gs* (mol · m^−2^s^−1^)	*Ci* (μmol · CO_2_ mol^−1^)	*E* (mmol · m^−2^s^−1^)	*WUEi*
female	CK	18.59 ± 1.31 c	0.16 ± 0.01 abc	159.56 ± 5.75 de	3.46 ± 0.21 bc	5.37 ± 0.12 def
	W-90d	15.27 ± 0.38 de	0.13 ± 0.01 def	167.33 ± 12.96 bcd	2.93 ± 0.14 de	5.28 ± 0.30 ef
	W-140d	12.04 ± 0.99 f	0.15 ± 0.01 bcd	251.48 ± 12.02 a	3.21 ± 0.08 bcd	3.77 ± 0.36 h
	CK-R	21.30 ± 0.41 b	0.18 ± 0.01 a	180.04 ± 10.38 bcd	3.84 ± 0.13 a	5.60 ± 0.28 cde
	W-90d-R	24.06 ± 0.59 a	0.15 ± 0.00 cde	115.89 ± 8.29 f	3.14 ± 0.06 cd	7.67 ± 0.25 b
	W-140d-R	22.00 ± 0.98 ab	0.12 ± 0.01 fg	84.91 ± 11.86 g	2.22 ± 0.15 f	10.00 ± 0.42 a
male	CK	16.30 ± 0.53 d	0.17 ± 0.01 ab	195.83 ± 5.80 bc	3.93 ± 0.18 a	4.17 ± 0.13 gh
	W-90d	15.52 ± 0.79 de	0.11 ± 0.01 g	148.42 ± 14.11 de	2.38 ± 0.09 f	5.84 ± 0.35 cde
	W-140d	13.85 ± 0.45 ef	0.15 ± 0.01 bcd	198.68 ± 7.27 b	3.32 ± 0.09 bcd	4.70 ± 0.21 fg
	CK-R	21.04 ± 0.46 b	0.15 ± 0.00 bcd	168.46 ± 6.63 bcd	3.30 ± 0.11 bcd	6.41 ± 0.23 c
	W-90d-R	22.09 ± 0.76 ab	0.17 ± 0.00 abc	164.99 ± 10.28 cde	3.56 ± 0.05 ab	6.22 ± 0.24 cd
	W-140d-R	21.34 ± 0.74 b	0.13 ± 0.00 efg	133.82 ± 13.06 ef	2.59 ± 0.07 ef	8.29 ± 0.39 b
*F*_*S*_		0.201	0.570	0.139	0.960	0.464
*F*_W_		0.769	0.740	0.508	0.983	0.881
*F*_R_		0.000	0.895	0.756	0.623	0.001
*F*_S×W_		0.000	0.000	0.000	0.000	0.000
*F*_S×R_		0.001	0.191	0.015	0.051	0.000
*F*_W×R_		0.000	0.000	0.000	0.000	0.000
*F*_S×W×R_		0.000	0.000	0.000	0.000	0.000

CK, control treatment; W, flooding and waterlogging stress treatment; R, recovery after waterlogging stress. Values are means ± SE (*n* = 5). Letters above the columns indicate significantly differences at *P* < 0.05 according to Duncan’s test. The significance values of the factorial analysis (ANOVA) for: *F*_*S*_, *F*_W_, and *F*_R_ refer different effects of species, watering, and recovery growth, respectively; *F*_S×W_, *F*_S×R_, *F*_W×R_, and *F*_S×W×R_ refer species × watering effects, species × recovery growth effects, watering × recovery growth effects, and species × watering × recovery growth effects, respectively.

**Table 2:** Pigment content variation in male and female *P. deltoides* under winter flooding stress and post-flooding recovery.

Species	Treatment	*Chl a* (mg/g· FW)	*Chl b* (mg/g· FW)	*Caro* (mg/g· FW)	*Total Chl* (mg/g· FW)	*Ch a*/*Ch b* (mg/g· FW)
female	CK	0.57 ± 0.11 cde	0.05 ± 0.01 de	0.02 ± 0.01 cd	0.62 ± 0.11 cde	11.40 ± 2.60 abc
	W-90d	0.34 ± 0.03 de	0.04 ± 0.01 e	0.01 ± 0.00 d	0.31 ± 0.03 e	8.53 ± 1.94 bc
	W-140d	0.27 ± 0.02 e	0.04 ± 0.00 e	0.01 ± 0.00 d	0.38 ± 0.02 e	7.07 ± 0.45 c
	CK-R	1.58 ± 0.04 ab	0.12 ± 0.01 bc	0.11 ± 0.02 ab	1.70 ± 0.05 ab	13.94 ± 1.34 ab
	W-90d-R	0.95 ± 0.20 c	0.10 ± 0.01 cd	0.03 ± 0.00 cd	1.00 ± 0.21 cd	9.24 ± 1.39 bc
	W-140d-R	0.90 ± 0.16 c	0.12 ± 0.01 bc	0.03 ± 0.01 cd	1.07 ± 0.17 c	8.12 ± 1.01 bc
male	CK	0.73 ± 0.03 cd	0.04 ± 0.01 e	0.05 ± 0.01 cd	0.77 ± 0.02 cde	17.35 ± 2.90 a
	W-90d	0.51 ± 0.10 cde	0.05 ± 0.00 de	0.03 ± 0.01 cd	0.56 ± 0.10 de	10.10 ± 1.58 bc
	W-140d	0.43 ± 0.14 de	0.04 ± 0.00 e	0.03 ± 0.03 cd	0.47 ± 0.14 e	10.27 ± 3.67 bc
	CK-R	1.97 ± 0.06 a	0.15 ± 0.01 ab	0.13 ± 0.01 a	2. 12 ± 0.06 a	13.04 ± 0.82 abc
	W-90d-R	1.83 ± 0.30 ab	0.18 ± 0.03 a	0.08 ± 0.02 abc	2.01 ± 0.34 ab	10.75 ± 1.35 bc
	W-140d-R	1.48 ± 0.20 b	0.11 ± 0.00 bc	0.07 ± 0.03 bc	1.60 ± 0.19 b	12.98 ± 1.64 abc
*F*_*S*_		0.467	0.620	0.532	0.476	0.275
*F*_W_		0.021	0.461	0.326	0.020	0.073
*F*_R_		0.000	0.000	0.000	0.000	0.709
*F*_S×W_		0.028	0.508	0.254	0.025	0.128
*F*_S×R_		0.000	0.000	0.003	0.000	0.305
*F*_W×R_		0.000	0.000	0.115	0.000	0.766
*F*_S×W×R_		0.000	0.000	0.000	0.000	0.050

CK, control treatment; W, flooding and waterlogging stress treatment; R, recovery after waterlogging stress. Values are means ± SE (*n* = 5). Letters above the columns indicate significantly differences at *P* < 0.05 according to Duncan’s test. The significance values of the factorial analysis (ANOVA) for: *F*_*S*_, *F*_W_, and *F*_R_ refer different effects of species, watering, and recovery growth, respectively; *F*_S×W_, *F*_S×R_, *F*_W×R_, and *F*_S×W×R_ refer species × watering effects, species × recovery growth effects, watering × recovery growth effects, and species × watering × recovery growth effects, respectively.

Winter flooding durations had little effects on majority physiological parameters in both sexes of *P. deltoides*. For example, *Chl a*, *Chl b*, *Caro*, *Total Chl*, *Chl a*/*Chl b*, *qP*, *qN*, REL, RWC, GSH, soluble protein, reducing sugar, proline, MDA, ·OH, O_2_^.−^ (Fig. 5D), POD, SOD, CAT, and GR in both sexes of *P. deltoides* did not vary significantly between medium flooding stress (W-90d) and severe winter flooding stress (W-140d). However, significant variations in *Ci* (Table 1) and *WUEi* were found in both sexes of *P. deltoides* between W-90d and W-140d treatments. In addition, there were significant variations in some parameters, including *A*, *gs*, *E*, *Fv/Fm*, *Yield*, *ETR*, H_2_O_2_, and APx, but only in either females or males (Supplementary Table 1).

When the flood water drained away, *Chl b, Chl a*/*Chl b*, *A*, *WUEi*, *Fv/Fm*, *Yield*, *ETR*, *qP*, *qN*, REL, RWC, GSH, soluble protein, proline, H_2_O_2_, MDA, ·OH, O_2_^.−^, POD, SOD, CAT, and GR in previously severe flooded females and males of *P. deltoides* after 15 d recovery growth (W-140d-R) recovered to normal or even better levels than in individual unflooded plants (Supplementary Table 1). In addition, previously flooded plants subjected to different flooding durations (W-90d-R and W-140d-R) exhibited insignificant differences in terms of *A*, *Chl a*, *Caro*, *Total Chl*, *Chl a*/*Chl b*, *Fv/Fm*, *Yield*, *ETR*, *qP*, *qN*, RWC, REL, GSH, soluble protein, reducing sugar, proline, H_2_O_2_, MDA, OH, O_2_^.−^, POD, SOD, CAT, and GR after 15 days of recovery growth after flood water was drained away.

### Sexually-dependent physiological responses to winter flooding combined with waterlogging stress, and post-flooding recovery

There were no significant intersexual differences in terms of *A, gs, E, Chl a*, *Chl b*, *Caro*, *Total Chl*, *Chl a* /*Chl b*, *Yield, ETR*, *qP*, REL, GSH, soluble protein, reducing sugar, proline, ·OH, POD, SOD, CAT, and GR under severe winter flooding conditions (W-140d), and severe winter flooding stresses had almost equal effects on males and females in terms of *gs, E, Chl b*, *Caro*, *Total Chl*, *qP*, REL, GSH, reducing sugar, and POD. However, although significant declines in *A*, *Fv/Fm*, *Yield*, and *ETR* were found in both sexes of *P. deltoides* under severe winter flooding stress condition (W-140d), significant variations in *A*, *Fv/Fm*, *Yield*, and *ETR* (Supplementary Table 1) under medium winter flooding stress conditions (W-90d) were only found in females, but not in males.

Interestingly, the sexes did differ in terms of H_2_O_2_, MDA, O_2_^.−^, OH, APx, and CAT (Supplementary Table 1). Under severe flooding conditions (W-140d), significant increases in MDA and O_2_^.−^, and increases in OH, were observed in females, while significant decreases in O_2_^.−^, and decreases in ·OH, and insignificant variation in MDA were observed in males. In addition, compared with flooded males, the flooded females had higher levels of H_2_O_2_, O_2_^.−^, and MDA. Although both sexes of *P. deltoides* exposed to winter flooding stress displayed similar trends and activity levels of POD, SOD, and GR, there were differences in the trends of APx and CAT activities (Supplementary Table 1). Males showed significant increases in APx activity but no variation in CAT activity. In contrast, female APx activity did not vary, while CAT activity decreased.

In general, previously-flooded females and males had similar recovery capabilities in terms of majority physiological parameters during the post-recovery stage. However, when the flood water drained away, males showed significant higher levels in *Ci*, *E*, *Chl a*, *Chl b*, and *Total Chl*, and significant lower levels in *WUEi* and MDA in comparison to females under W-90d-R conditions. In addition, compared with females, males showed significant higher levels in *Ci*, *E*, *Chl a*, *Total Chl*, and APx, and significant lower levels in *WUEi* and MDA under W-140d-R conditions.

### Comparative analysis of ultrastructural morphology

**Figure 7:**
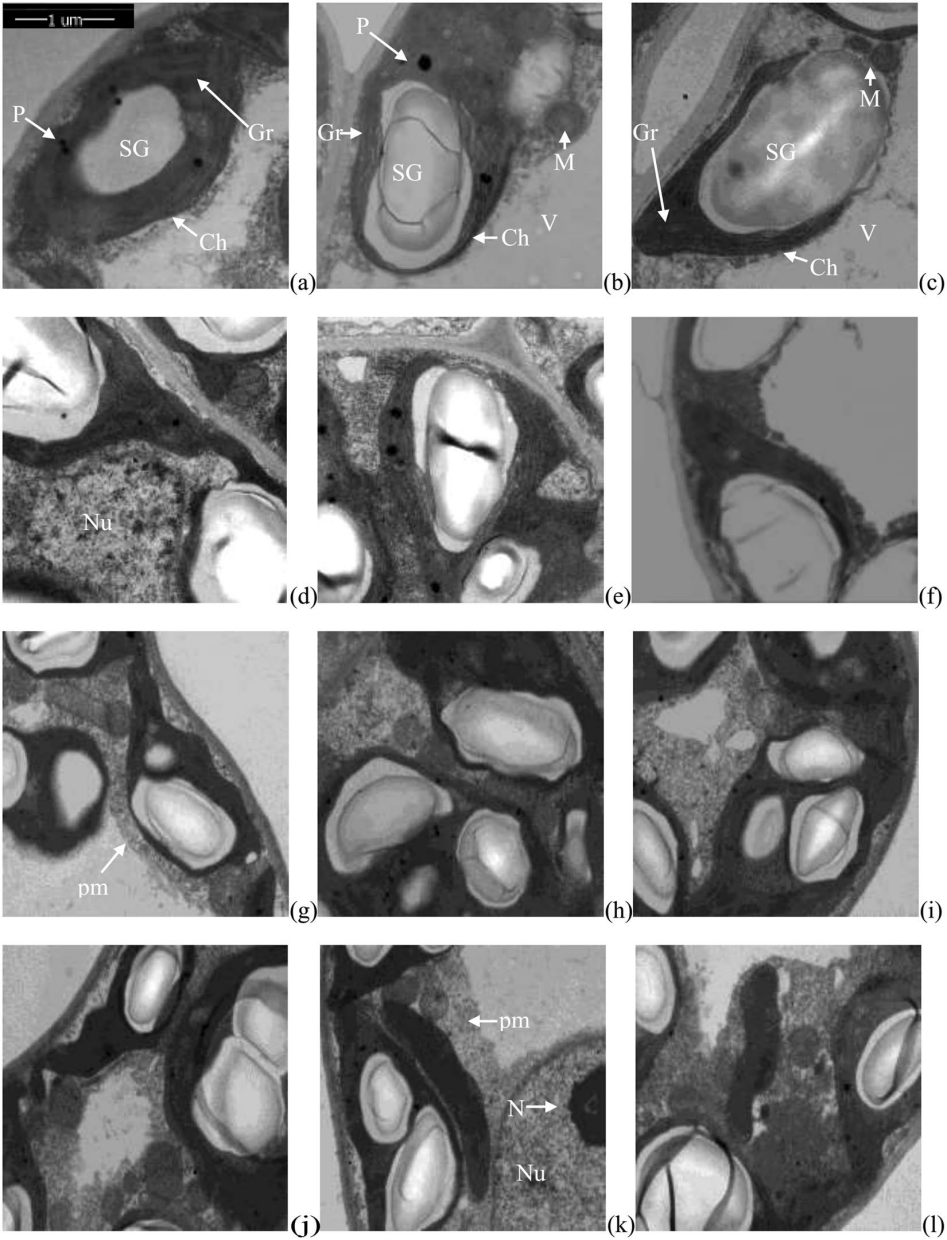
Ultrastructural variations in male and female *P. deltoides* under winter flooding stress and post-flooding recovery. CK, control treatment; W, flooding and waterlogging stress treatment; R, recovery after waterlogging stress. (**a**) CK females; (**b**) W-90d females; (**c**) W-140d females; (**d**) CK males; (**e**) W-90d males; (**f**) W-140d males; (**g**) CK-R females; (**h**) W-90d-R females; (**i**) W-140d-R females; (**j**) CK-R males; (**k**) W-90d-R males; (**l**) W-140d-R males. Ch, chloroplast; Gr, granum; M, mitochondrion; N, nucleolus; Nu, nucleus; P, plastoglobule; SG, starch grain; V, vacuole.

We next asked how the ultrastructural morphology of *P. deltoides* females and males varied in response to winter flooding stress and post flooding recovery. Thus, we examined their cellular ultrastructure. As shown in Fig. 7, chloroplasts of both sexes were well arranged. The majority had distinct granum regions, and some small plastoglobules were found, especially in females. Under medium flooding stress (W-90d), plastoglobules became enlarged in both sexes, and their numbers decreased in females. However, under severe flooding stress (W-140d), disintegrated chloroplasts and numerous tilted granal stacks were found, and plastoglobules gradually disappeared. Some large vesicles, starch grains, and swollen mitochondria were found, especially in females. With the growth of development, more distinct granum and small starch grains developed. During the post-flooding recovery stage, the percentage of vesicles decreased, while distinct granum regions increased. The number of mitochondria, small starch grains, and small plastoglobules increased. In addition, no significant differences in the ultrastructural morphology of *P. deltoides* females and males were observed.

## Discussion

Winter flooding is a special event in some rivers due to the artificial water level regulation. Kozlowski^3^ suggested that flooding during the growing season can have greater negative effects on deciduous species than flooding during the dormant season. However, information about morphological and physiological responses to winter flooding stress is still scarce. Therefore, some researchers have suggested that the mechanisms of winter flooding adaptation in flooding tolerant species should be investigated to better understand their survival responses to reversed flooding patterns^1–3^

All plants survived 140 days of winter flooding without mortality, leaf chlorosis, leaf necrosis, or leaf abscission, which suggests that, in terms of seedling survival, both female and male *P. deltoides* are winter flood tolerant. Therefore, both female and male *P. deltoides* are superior candidates for the construction of protective riparian forests in winter flooding areas. Flooding or waterlogging during the growing season can induce aerenchyma tissues and adventitious roots in a wide variety of both flood-intolerant and tolerant angiosperms and gymnosperms, especially in flood tolerant species, as concluded by Kozlowski^3^. However, some often-visible morphological adaptations, including the formation of hypertrophied lenticels, aerenchyma tissues and adventitious roots, were not observed in winter flooding-stressed female and male *P. deltoides*, which might be a result of low oxygen demands during the dormant season.

The majority of previous studies have demonstrated that flooding or waterlogging stresses can decrease plant growth and development, pigment content, photosynthetic capacity, stomatal conductance, and chlorophyll fluorescence. They may also increase ROS and MDA levels, increase the contents of some cellular non-enzymatic components, and increase the activity of antioxidant enzyme systems^2–4, 6–8, 23, 24^. Compared with summer waterlogging during growth and development, winter flooding stress during the dormant season caused fewer physiological dysfunctions in *P. deltoides* ^5, 6^. The duration of flooding significantly affects survival rates, plant growth and yield, and physiological responses^1, 3, 9^. Our results suggest that female and male of *P. deltoides* were not very sensitive to flooding duration. The duration of winter flooding had little effect on plant growth, physiology, and biochemistry due to the low levels metabolic activity occurring during the dormant season.

Normally, although the stomata reopen slowly and the rate of photosynthesis increases when flood water drains away, previously-flooded plants may have difficultly recovering to normal levels of growth, photosynthetic capacity, and other physiological responses, because absorption of water by their small root systems can’t adequately replenish transpirational losses within a short period^3, 25^. In present study, the ultrastructural morphology of mesophyll cells and majority physiological parameters in previously flooded plants recovered to normal after 15 d recovery growth. These phenomena may be because the winter flooding stress caused little damage to the one-year-old root systems, and then the previously flooded plants could absorb water and nutrients normally after flood water drained away. These results also suggest that *P. deltoides* has strong self-repairing capabilities during post-flooding recovery.

Some studies of trees have observed sex-specific responses to a number of biotic stresses^26, 27^ and environmental stresses such as waterlogging stress^6^, water deficit^16^, chilling^17^, salinity^21^, enhanced UV-B radiation^28^, atmospheric CO_2_ enrichment^29^, nutrient deficiency^19, 30^, excess manganese, and a combination of different stresses^31, 32^. These studies concluded that females seem to be more sensitive to environmental stress and usually experience greater negative effects. In other cases, female plants exhibited better tolerance of adverse conditions than males, females showed a more conservative strategy of water use or higher photosynthetic rates compared with males^4, 33–35^. For example, Male and female *P. angustifolia* genotypes grew similarly with favorable water levels, but males tended to be more inhibited by flooding, while females were more flood tolerant^4^. Females of some perennial herbs are more resistant to drought stress than males^35^. Therefore, the information available thus far is insufficient for establishing generalizations about whether male and female dioecious plants show differential abiotic stress resistance. Furthermore, most comparisons of male and female performance have mainly focused on short-term physiological measurements during the growth season. In this paper we highlight the need for physiological studies of sexual dimorphism in dioecious plants in response to different durations of winter flooding stress during the dormancy season and during post-flooding recovery.

Both sexes had similarly strong flooding tolerances in terms of the majority of physiological parameters and ultrastructural variations at an early stage of development. Differences between sexes do not always exist. For example, the female and male *P. angustifolia* did not display significant differences in photosynthetic gas exchange, leaf reflectance, chlorophyll fluorescence, and WUE in response to groundwater availability, and soil water typically declines between May and September^7^. The similar sexual responses of *P. deltoides* to winter flooding stress may be the result of low metabolic activity during the dormancy season and early growth developmental stage.

The significant variations in *A*, *Fv*/*Fm*, *Yield*, and *ETR* (Supplementary Table 1) only in females under medium winter flooding stress conditions (W-90d) suggest that female *P. deltoides* are more sensitive than males to winter flooding stress in terms of photosynthesis and chlorophyll fluorescence. Our results are consistent with previous studies. For example, female *P. yunnanensis* grown in China during a drought exhibited gas exchange rate depression and greater damage to cell organelles than male did^32^. Female *P. cathayana* grown in China were also more responsive and showed greater negative effects on net photosynthesis than males when grown under increased drought stress and elevated temperatures^16^. Additionally, although the previously-flooded females and males had similar recovery capabilities in the post-flood period, males had better recovery capabilities than females in terms of pigment content, especially *chl a*.

Root oxygen deficiency resulting from flooding stress caused photoxidative damage to leaves via increased generation of ROS^2, 3^. These are important signaling molecules indicative of oxidative stress. They can directly attack membrane lipids, resulting in lipid peroxidation and oxidation of proteins and nucleic acids^6, 36–38^. The major indicators of ROS accumulation are H_2_O_2_, O_2_^.−^, and ·OH. One of the most frequently-used indicators of lipid peroxidation is MDA, and MDA content reflects the degree of membrane lipid peroxidation^6, 37, 38^. The sexes differ in terms of H_2_O_2_, O_2_^.−^, OH, MDA, APx, and CAT in response to winter flooding stress, which suggest that female plants encountered more serious oxidative damage during winter flooding stress than males. Plants can protect cellular and sub-cellular systems to control ROS levels and membrane lipid peroxidation with antioxidant enzymatic systems. These results indicate that males develop more efficient antioxidant enzymatic systems to control ROS accumulation than females do. Our results indicate that, in terms of antioxidant enzymatic systems, *P. deltoides* males are more tolerant to winter flooding stress than females. Previous studies have shown that females of *P. yunnanensis*, *P. cathayana*, and *P. deltoides* exhibit greater ROS accumulation and oxidative stress damage, and have less efficient antioxidant enzymatic systems than males during environmentally stressful growth season conditions^6, 17, 18, 21, 28, 30^.

In conclusion, both sexes of *P. deltoides* are winter flood tolerant, based on seedling survival, and morphological, physiological, and ultrastructural responses. Winter flooding stress differentially affected physiological traits in *P. deltoides* at early growth stages of development. Significant variations in terms of *chl a*, *chl b*, *caro*, *total chl*, *chl a*/*chl b*, *gs*, soluble protein, reducing sugar, proline, ·OH, CAT, and POD were absent under severe winter flooding stress in both sexes. In addition, fatally-damaging ultrastructural responses were not found in *P. deltoides*. In both sexes, the duration of winter flooding stress had insignificant effects on morphological, ultrastructural, and the majority of physiological responses, except for *A*, *gs*, *E*, *Fv/Fm*, *Yield*, *ETR*, H_2_O_2_, and APx. When flood water was drained away, previously-flooded plants grew at a faster rate than unflooded plants. The majority of physiological parameters and ultrastructural morphology of mesophyll cells in previously-flooded plants could recover to normal levels in previously-flooded plants, whose recover was better than that of unflooded plants. Both sexes had similar responses to W-90d and W-140 conditions in terms of majortity physiological parameters. However, females were more sensitive than males to winter flooding stress in terms of photosynthesis and chlorophyll fluorescence, based on their significant declines in *A*, *Fv*/*Fm*, *Yield*, and *ETR* under W-90d conditions. Females encountered more serious oxidative damage than males under flooding conditions. The results indicate that males develop more efficient antioxidant enzymatic systems to control ROS accumulation than females. Additionally, although previously-flooded females and males had strong and similar post-flooding recovery capabilities, males recovered better in terms of pigment content, especially *chl a*. This study provides new light on the adaptation mechanisms of *P*. *deltoides* trees subject to winter flooding stress. It also increases the understanding of sexually-dimorphic responses to flooding stress during the dormant season, and to post-flooding recovery.

## Materials and Methods

### Plant materials and experimental design

One-year-old cuttings of *P. deltoides* were collected from 25 female and 25 male trees at Qianjiang (30°09′ N, 121°31′ E), Hubei Province, China. The cuttings were planted in March 2013. After sprouting and growing for about 2 months, 360 cuttings (180 females and 180 males) with similar crown sizes and equal heights were selected and each was replanted into a 10 L plastic bucket filled with 10 kg homogenized soil. Plants were placed in a natural environment with 1261 mm mean annual rainfall, 1494 mm annual evaporation, 80% annual relative humidity and 16.9 °C annual temperature at the Wuhan Botanical Garden, Chinese Academy of Sciences. After a growing season, the branches of all plants were pruned to an identical height, i.e., 10 cm above ground level, and the leaves were removed. These plants were used for subsequent winter flooding treatments.

**Figure 8:**
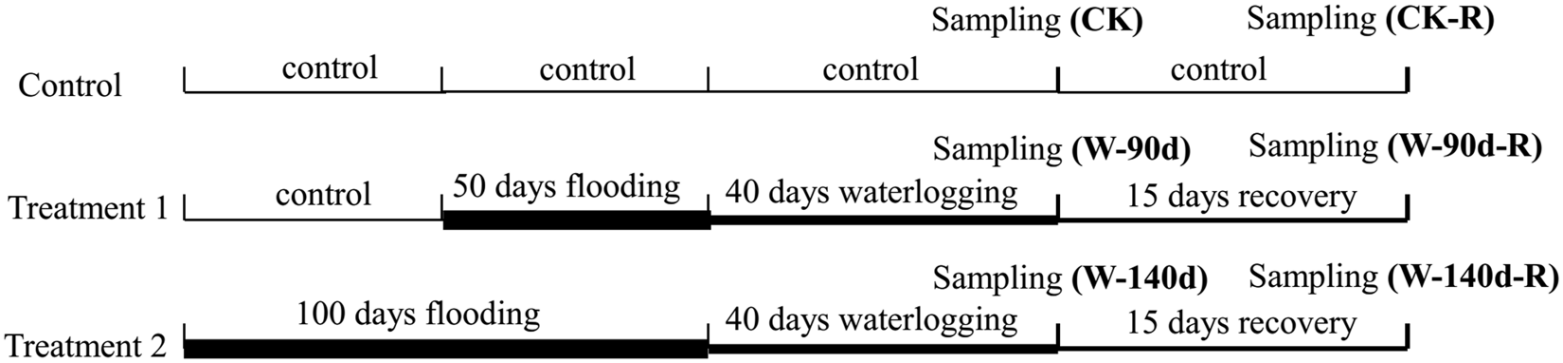
Illustration of the experimental design.

The experimental layout was completely randomized according to the two main factors (sex and watering rate; Fig. 8). Three watering treatment regimes were employed, being well-watered (CK), 100 days winter flooding stress combined with 40 days waterlogging (severe flooding stress, W-140d), and 50 days winter flooding stress combined with 40 days waterlogging treatment (medium flooding stress, W-90d). In the well-watered treatment, all pots were watered excessively every three days and excess water was allowed to drain through drainage holes into dishes placed under the buckets. In the winter flooding treatment, pots were watered every nine days to 5 cm above the top of the plants. For 100 days and 50 days winter-flooding treatments, the plants were submerged in December and in the next February, respectively. Both flooding treatments were simultaneously transferred to waterlogging treatments on March 29, 2014 for sprouting before the start of the growing season. During the next 40 days of waterlogging treatment, pots were watered every nine days to 5 cm above the soil surface (W-140d and W-90d). At the end of the waterlogging treatment, each plant’s shoot height, basal stem diameter, gas exchange rate and chlorophyll fluorescence were measured. Fresh leaves were collected for physiological analyses, and then the excess water was drained away on May 6, 2014. During following post recovery stage, all pots were watered excessively every three days for 15 days of growth recovery (CK-R, W-140d-R, and W-90d-R) as above description. At the end of the 15 day growth recovery period, each plant’s shoot height, basal stem, gas exchange rate and chlorophyll fluorescence were measured, and fresh leaves were collected for physiological analyses. All plants used for fresh leaf collection were not used in the next treatment. Five replications, each with six cuttings, were used for each treatment.

### The morphological traits

At the end of each experimental stage, the plant height and basal stem diameter of each tested plants were measured. The comparative observations on leaf senescence and abscission, hypertrophied lenticels, aerenchyma tissue, and adventitious roots were performed each week.

### Gas exchange measurements

The net photosynthetic rate (*A*), stomatal conductance (*gs*), intercellular CO_2_ concentration (*Ci*) and transpiration (*E*) were measured from 9:00 to 11:30 am on 5, 20 May with a LI-COR 6400 portable photosynthesis system (LI-COR Inc. Lincoln, Nebr.), respectively. The PAR, provided by a 6400-02 LED light source, was set to 1400 μmol m^−2^s^−1^. The flow rate of air through the sample chamber was set at 500 μmol m^−2^s^−1^, and the leaf temperature and relative humidity was maintained at 25 ± 0.8 °C by thermoelectric coolers and 50%, respectively. The methods are modified from Yang *et al*.^6^ and Xu *et al*.^16^. Five cuttings from each treatment were selected for measuring. A measurement was made on each of the three terminal leaflets of the uppermost fully opened leaf of each cutting. Instantaneous water use efficiency (WUEi = *A/E*) was calculated by dividing photosynthetic rate by transpiration.

### Determination of chlorophyll content

Chlorophylls were extracted in 80% (v/v) chilled acetone and quantified using a spectrometer (UV-1800PC, MAPADA, Shanghai) as described by Yang *et al*.^6^ and Xu *et al*.^16^. The absorbances of chlorophyll *a* (*Chl a*), chlorophyll *b* (*Chl b*), and carotenoids (*Caro*) were determined at 663 nm, 646 nm, and 470 nm, respectively. The absorbance values were converted to concentrations as described by Lichtenthaler^39^. And the total chlorophyll (chlorophyll *a* + *b*, *Total Chl*) and *Chl a*/*Chl b* were calculated.

### Chlorophyll fluorescence measurements

We selected the same five cuttings and same leaves that used for gas exchange measurements for chlorophyll fluorescence measurements. Chlorophyll fluorescence kinetics parameters (*F*v/*F*m, maximum efficiency of PSII; *Yield*, the effective quantum yield of PSII; *qN*, non-photochemical quenching coefficient; *qP*, photochemical quenching coefficient; *ETR*, photosynthetic electron transportation rate) were measured with a PAM chlorophyll fluorometer (PAM 2500, Walz, Effeltrich, Germany). The leaf samples were placed in darkness for 30 min by covering with aluminum foil followed by measurement of minimum fluorescence (*F*o) at 250 μmol m^−2^s^−1^ PPF and *F*m at 2400 μmol m^−2^s^−1^ PPF following a saturating pulse of actinic light^6, 11^. Measurements were carried out between 8:30 and 11:30 on May 6 and 21, respectively.

### Determination of relative water content and relative electrolyte leakage

The fourth-sixth fully expanded leaves were sampled to determine the leaf relative water content (RWC) as described by Yang *et al*.^6^. Five freshly cut leaf discs (1.5 cm in diameter) from the fifth fully expanded leaves were used determined the leaf relative electrolyte leakage (REL) using a conductivity instrument (FE38, Mettler-Toledo Instruments Co., Ltd, Shanghai, China) according to procedure of Zhang *et al*.^17^.

### Determination of soluble protein content, glutathione (GSH), reducing sugar, and free proline content

About 2 g fresh samples were ground with liquid nitrogen and then homogenized in 10 ml 100 mM universal sodium phosphate extraction buffer as described by Han *et al*.^40^. The supernatant was stored in volumes of 0.5 ml at –80 °C until using for the determination of soluble protein, reactive oxygen species (ROS) level and antioxidant enzymes activities^40^. The soluble protein was quantified by Bradford method^41^, the soluble protein content was expressed as mg/g · FW.

The concentrations of GSH, reducing sugar, proline were assayed according the procedure of Bates *et al*.^42^, Sairam *et al*.^43^ and Yang *et al*.^6^. The GSH concentration, reducing sugar, and proline concentration were calculated and expressed as mg/g·FW, mg/g·FW, and μg/g·FW, respectively.

### Determination of ROS level and malondialdehyde (MDA) content

The detections of superoxide (O_2_^.−^), hydrogen peroxide (H_2_O_2_), hydroxyl radicals (·OH), and MDA were based on the procedures of Yang *et al*.^6^ and Yang *et al*.^38^. The concentrations of O_2_^.−^, H_2_O_2_, ·OH, and MDA were calculated and expressed as ng/g·FW, μmol/g·FW, ng/g·FW, and μmol/g·FW, respectively.

### Assay of antioxidant enzymes activities

The antioxidant enzyme including guaiacol peroxidase (POD), ascorbate peroxidase (APx), catalase (CAT), superoxide dismutase (SOD), and glutathione reductase (GR) activities were determined according to the manufacturer’s instructions as described Zhang *et al*.^17^ and Han *et al*.^40^. The activities of POD, CAT, and GR were calculated and expressed as U/mg·protein, The APx activities were determined as described by Yang *et al*.^38^, and expressed as U/g·protein. The CAT activity was calculated and expressed as U/mg · protein.

### Transmission electron microscopy

Three small leaf sections (2 cm in length, 1 cm in width) from fifth fully expanded leaves, avoiding the midrib, were selected for the transmission electron microscope analysis according the procedures of Zhang *et al*.^17^. The sections were fixed in 2.5% (v/v) glutaral pentanedial in 0.2 M of PBS (sodium phosphate buffer, pH 7.0) for 3 h at 25 °C and postfixed in 2% osmium tetraoxide (OsO4) for 2 h. The tissues were then sequentially dehydrated in 30%, 50%, 70%, and 90% acetone, and embedded in Epon 812 for 2 h. Ultra-thin sections (80 nm) were sliced, stained with uranyl acetate and lead citrate, and mounted on copper grids for viewing in the H-7000FA TEM (Japan) at an accelerating voltage of 160 kV.

### Statistical analyses

Results were expressed as means ± standard errors (n = 5). SPSS 13.0 software was used for statistical analysis. Analyses of variance (ANOVA) for variables from measurements were used for testing the species and treatment differences. Differences were considered significant at P < 0.05.

## Electronic supplementary material

Supplementary Table 1
