# Supplementary material for: Sex-specific responses to winter flooding, spring waterlogging and post-flooding recovery in Populus deltoides
Source: Sci Rep. 2017 May 31;7:2534. doi: 10.1038/s41598-017-02765-2 (PMC5451430; doi:10.1038/s41598-017-02765-2)
Supplement: Supplementary file 1 — Supplementary Table 1 [file 41598_2017_2765_MOESM1_ESM.doc]

**Sex-specific responses to winter flooding, spring waterlogging and post-flooding recovery in *Populus deltoides***

Ling-Feng Miao, Fan Yang^*^, Chun-Yu Han, Yu-Jin Pu, Yang Ding, Li-Jia Zhang

Institute of Tropical Agriculture and Forestry, Hainan University, Haikou, Hainan, 570228, P. R. China

* Correspondence address:

Fan Yang (Email: [yangfan@hainu.edu.cn](mailto:yangfan@hainu.edu.cn); [fanyangmlf6303@163.com](mailto:fanyangmlf6303@163.com))

Supplementary 1 The index of comparative analysis on differences. CK, control treatment; W, flooding and waterlogging stress treatment; R, recovery after waterlogging stress. Values followed by different little letters (Fig. 2-9) are significantly different at according to Duncan’s test (P < 0.05). *, significant difference (P < 0.05), followed according to the absolute different letters between two comparative parameters; +, one is higher than another, followed according to part different letters between two comparative parameters; —, one is lower than another, followed according to part different letters between two comparative parameters; =, no variation, followed according to completely same letters between two comparative parameters.

|  | At early growth stage (First sampling) | | | | | | | | | At recovery stage (Second sampling) | | | | | | | | |
| --- | --- | --- | --- | --- | --- | --- | --- | --- | --- | --- | --- | --- | --- | --- | --- | --- | --- | --- |
| **Traits** | Well- watered (CK) | Medium flooding  (W-90d vs CK) | | | Severe flooding  (W-140d vs CK) | | | W-140d vs W-90d | | Well-  watered (CK-R) | Medium flooding recovery (W-90d-R vs CK-R) | | | Severe flooding recovery (W-140d-R vs CK-R) | | | W-140d-R  vs W-90d-R | |
|  | Female vs Male | Female | Male | Female vs Male | Female | Male | Female vs Male | Female | Male | Female vs Male | Female | Male | Female vs Male | Female | Male | Female vs Male | Female | Male |
| **Table 1** |  |  |  |  |  |  |  |  |  |  |  |  |  |  |  |  |  |  |
| ***A*** | *+ | *— | — | = | *— | *— | — | *— | — | = | *+ | + | + | + | = | — | — | — |
| ***g_s_*** | — | *— | *— | *+ | — | — | = | + | *+ | *+ | *— | + | — | *— | *— | — | *— | *— |
| ***Ci*** | *— | + | *— | + | *+ | + | *+ | *+ | *+ | = | *— | — | *— | *— | *— | *— | *— | — |
| ***E*** | *— | *— | *— | *+ | — | *— | = | + | *+ | *+ | *— | + | *— | *— | *— | — | *— | *— |
| ***WUEi*** | *+ | — | *+ | — | *— | + | *— | *— | *— | — | *+ | — | *+ | *+ | *+ | *+ | *+ | *+ |
| **Table 2** |  |  |  |  |  |  |  |  |  |  |  |  |  |  |  |  |  |  |
| ***Chl a*** | — | — | — | — | — | — | — | — | — | — | *— | — | *— | *— | *— | *— | = | — |
| ***Chl b*** | + | — | + | — | — | = | = | = | — | — | — | + | *— | = | — | = | + | *— |
| ***Caro*** | = | — | = | — | — | = | = | = | = | — | *— | — | — | *— | *— | — | = | — |
| ***Total Chl*** | = | — | — | — | — | — | = | = | — | — | *— | — | *— | *— | *— | *— | + | — |
| ***Chl a / Chl b*** | — | — | *— | — | — | — | — | — | = | + | — | — | = | — | = | — | = | + |
| **Fig. 1** |  |  |  |  |  |  |  |  |  |  |  |  |  |  |  |  |  |  |
| **Shoot height** | = | *— | *— | = | *— | *— | = | = | = | = | *— | *— | = | *— | *— | = | = | = |
| **Basal Stem** | = | *— | *— | = | *— | *— | = | — | = | = | *— | *— | = | *— | *— | = | = | = |
| **Fig. 2** |  |  |  |  |  |  |  |  |  |  |  |  |  |  |  |  |  |  |
| ***Fv/Fm*** | *+ | *— | = | *— | *— | *— | *+ | = | *— | — | + | = | = | *+ | + | = | + | + |
| ***Yield*** | + | *— | — | = | *— | *— | + | — | *— | = | = | = | = | = | = | = | = | = |
| ***ETR*** | + | *— | = | — | *— | *— | + | = | *— | = | = | = | = | = | = | = | = | = |
| ***qP*** | *+ | — | + | = | *— | — | = | — | — | + | — | + | — | — | — | = | — | — |
| ***qN*** | = | = | + | — | + | *+ | *— | + | + | — | + | — | + | *+ | + | + | + | + |
| **Fig. 3** |  |  |  |  |  |  |  |  |  |  |  |  |  |  |  |  |  |  |
| **REL** | + | + | *+ | — | *+ | *+ | = | + | + | = | = | = | = | = | = | = | = | = |
| **RWC** | *+ | = | *— | *+ | — | *— | + | — | = | = | = | = | = | = | = | = | = | = |
| **Fig. 4** |  |  |  |  |  |  |  |  |  |  |  |  |  |  |  |  |  |  |
| **GSH** | = | *+ | *+ | = | *+ | *+ | = | + | + | *— | + | *— | + | = | — | — | — | + |
| **Soluble protein** | = | = | — | + | = | — | + | = | = | *+ | — | + | = | — | + | = | = | = |
| **Reduc.Sugar** | + | — | = | = | — | — | = | — | — | — | — | — | — | — | *— | — | — | — |
| **Proline** | + | = | — | + | — | — | + | — | = | — | + | + | + | *+ | *+ | + | + | + |
| **Fig. 5** |  |  |  |  |  |  |  |  |  |  |  |  |  |  |  |  |  |  |
| **H_2_O_2_** | + | *+ | *+ | *+ | *+ | *+ | *+ | *+ | = | = | = | = | = | = | = | = | = | = |
| **MDA** | + | + | = | *+ | *+ | + | *+ | + | + | *+ | + | + | *+ | + | + | *+ | + | = |
| **·OH** | *— | + | — | = | + | — | + | + | = | — | + | — | + | + | = | = | = | + |
| **O_2_^·-^** | *— | + | *— | *+ | *+ | *— | *+ | + | = | + | *— | — | *— | *— | = | *— | + | + |
| **Fig. 6** |  |  |  |  |  |  |  |  |  |  |  |  |  |  |  |  |  |  |
| **POD** | = | + | *+ | — | *+ | *+ | = | + | = | + | = | — | = | = | — | = | = | = |
| **SOD** | *— | *+ | + | *— | *+ | *+ | — | + | + | — | + | + | — | + | + | — | = | = |
| **APx** | *+ | = | + | + | + | *+ | *— | = | *+ | *+ | *— | + | — | *— | — | *— | *— | — |
| **CAT** | *+ | *— | = | = | — | = | + | + | = | *— | + | — | = | + | — | = | = | = |
| **GR** | + | — | — | = | — | — | + | = | — | = | + | = | = | = | = | — | — | = |
